# Bone single photon emission computed tomography with computed tomography disclosing chronic uterine perforation with intrauterine device migration into the anterior wall of the bladder: a case report

**DOI:** 10.1186/1752-1947-7-154

**Published:** 2013-06-10

**Authors:** David Morland, Carole Mathelin, Arnaud Wattiez, Izzie Jacques Namer

**Affiliations:** 1 Service de Biophysique et Médecine Nucléaire, Hôpitaux Universitaires de Strasbourg, Hôpital de Hautepierre, 1 avenue Molière, 67098, Strasbourg, Cedex 09, France; 2 Unité de Sénologie, Pôle de Gynécologie-Obstétrique, Hôpitaux Universitaires de Strasbourg, Hôpital de Hautepierre, 1 avenue Molière, 67098, Strasbourg, Cedex 09, France; 3 Service de Gynécologie, Pôle de Gynécologie-Obstétrique, Hôpitaux Universitaires de Strasbourg, Hôpital de Hautepierre, 1 avenue Molière, 67098, Strasbourg, Cedex 09, France; 4 ICUBE, Université de Strasbourg/CNRS UMR 7357, FMTS – Faculté de Médecine, 4 rue Kirschleger, 67085, Strasbourg, Cedex, France

**Keywords:** Intrauterine device complication, Bone scan, SPECT/CT

## Abstract

**Introduction:**

Extraosseous uptake of ^99m^Tc-hydroxymethylene diphosphonate is a common situation of variable clinical relevance.

**Case presentation:**

A 52-year-old Caucasian woman presented to our department for breast cancer staging. A ^99m^Tc-hydroxymethylene diphosphonate bone scan was performed and showed focal pelvic hyperfixation that disclosed intrauterine device migration into the anterior wall of the bladder on single photon emission computed tomography with computed tomography.

**Conclusion:**

This observation confirms the major role of single photon emission computed tomography with computed tomography in achieving an exact diagnosis.

## Introduction

Extraosseous soft tissue uptake of ^99m^Tc-hydroxymethylene diphosphonate (HMDP) is a common situation but of variable clinical relevance. These lesions are often encountered as incidental findings and may sometimes change the therapeutic approach for certain patients. The intrauterine device (IUD) is a frequently used and effective contraceptive method. Complications after IUD insertion, such as chronic uterine perforation, are rare but serious.

We present a case of a patient with chronic uterine perforation with IUD migration into the anterior wall of the bladder, discovered on bone single photon emission computed tomography with computed tomography (SPECT/CT).

## Case presentation

A 52-year-old Caucasian woman was referred to the Breast Diseases Center of the Strasbourg University Hospitals for the management of a bifocal infiltrating lobular carcinoma located in the upper outer quadrant of her right breast. She was multiparous (three children) and had been menopausal for one year. Her medical history showed no relevant data except for a Cesarian section. An IUD had been inserted after the last pregnancy 12 years ago. Another hormonal IUD had been inserted five years ago and removed before her breast surgery. A recent clinical pelvic examination had not revealed an IUD string. After neoadjuvant chemotherapy, she underwent a lumpectomy and axillary clearance. The histological examination revealed a residual focus of lobular carcinoma (pT1c) and each of the 30 axillary lymph nodes removed was negative. Breast surgery was followed by locoregional radiotherapy and hormonal therapy. A chest X-ray and an abdominal ultrasound for cancer staging were performed and revealed no metastasis. Cancer antigen (CA 15-3) showed a normal value (23U/ml).

**Figure 1:**
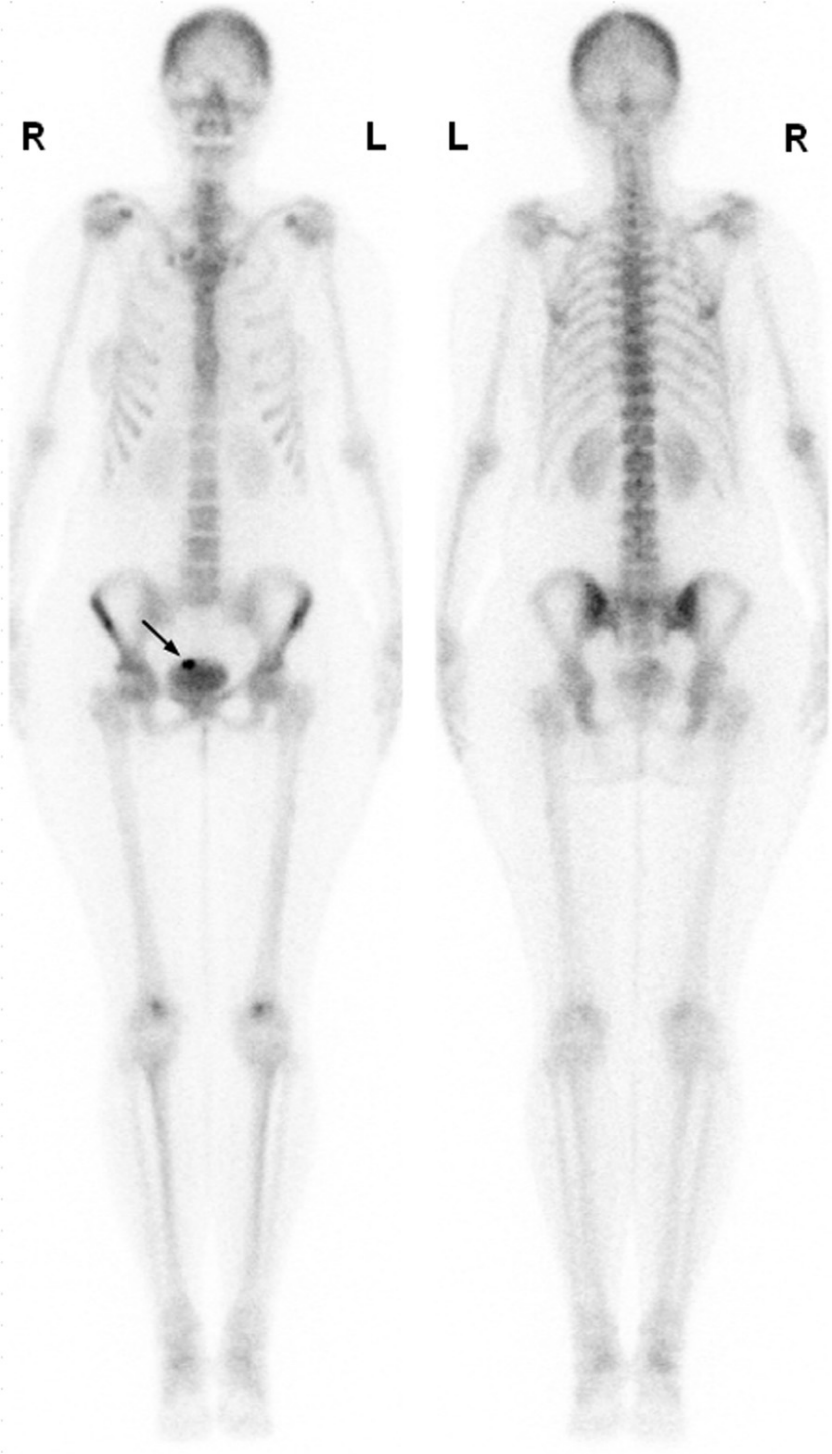
**Whole-body bone scan (anterior and posterior views) shows symmetrical and normal uptake of ^99m^Tc-hydroxymethylene diphosphonate throughout the skeleton.** However, there is a hot spot in the region of the bladder (arrow).

**Figure 2:**
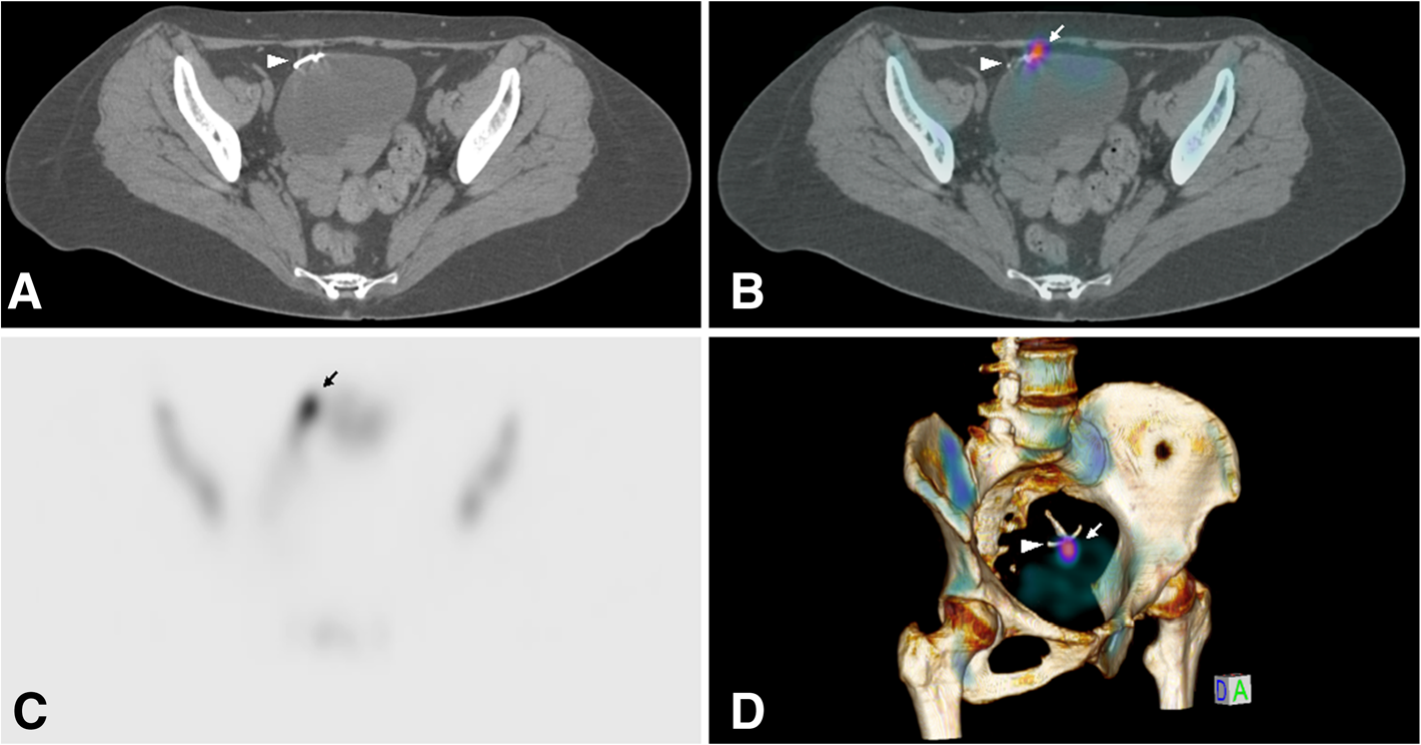
**Pelvic single photon emission computed tomography with computed tomography reveals a focal ^99m^Tc-hydroxymethylene diphosphonate hyperfixation (plain arrow) centered at a mislocated intrauterine device (arrow head) in the anterior vesical wall. (A)** Axial computed tomography slice. **(B)** Axial single photon emission computed tomography. **(C)** Axial single photon emission computed tomography with computed tomography. **(D)** Volume-rendering single photon emission computed tomography with computed tomography.

A bone scan for cancer staging was also performed in November 2012 (Figure 1). It showed symmetrical and homogeneous uptake of the tracer compound in bone structures. However, bone scintigraphy disclosed a focal pelvic hyperfixation in her bladder area on anterior incidence. SPECT/CT was performed and showed a mislocated IUD in the anterior vesical wall (Figure 2) corresponding to focal ^99m^Tc-HMDP hyperfixation due to a probable inflammatory granuloma surrounding the IUD.

## Discussion

Uterine perforation is a rare yet serious event and occurs at a rate between 1 out of 10,000 and 1 out of 350 IUD insertions [1]. In contrast to acute uterine perforation, which is often symptomatic and occurs during insertion, chronic perforation may occur at any time and is generally asymptomatic [2]. Several risk factors have been identified and can be classified into three categories [2-4]: (i) uterine factors: myometrial weakness (multiparity, Cesarean section), pronounced retro- or anteversion, uterine hypoplasia, insertion in the early post-partum period; (ii) IUD insertion technique including operator experience; and (iii) IUD type: perforation might be more frequent with copper IUDs [1].

Transuterine migration consists of two steps: uterine infraction and uterine migration. Spontaneous uterine contractions and local inflammation are found in these cases.

Migration usually occurs to the peritoneal cavity or to adjacent neighboring organs. In a review of the literature, we found 140 cases of intra-abdominal migration and 50 cases of intravesical migration of which 42.9 percent presented with lithiasis [2,4,5]. Indeed, calcification may occur when the IUD migrates into the bladder. However, there is no correlation between calcification and time spent in the bladder [3,4].

Mechanisms of extraosseous fixation are multiple and often intricate. When lesions contain microcalcification, the mechanism for increased accumulation is considered similar to that which occurs in normal bone: chemisorption onto calcium [6]. In injured tissue, intracellular calcium influx and calcium precipitation are responsible for the fixation.

Several diagnoses may be suspected when a pelvic hyperfixation occurs on a bone scan [7-9]. Typical causes are fourfold:

– Focal hyperfixation due to urinary tract pathology such as vesical diverticulum, calcified foreign body granuloma, bilharziasis or tuberculosis;

– Genital tract pathology: calcified uterine fibroid, endometrial tumor and a variety of ovarian tumors;

– Peritoneal metastasis;

– General causes: postradiation lesions, hematoma.

## Conclusions

IUD migration is a rare cause of pelvic extraosseous accumulation of bone radiotracers, which should be kept in mind.

This observation also confirms the major role played by SPECT/CT in achieving an exact diagnosis.

## Abbreviations

SPECT/CT: single photon emission computed tomography with computed tomography; IUD: intrauterine device.

## Competing interests

The authors declare that they have no competing interests.

## Authors’ contributions

DM and IJN diagnosed and drafted the manuscript. CM and AW followed up and managed the patient. All authors read and approved the final manuscript.

## Consent

Written informed consent was obtained from the patient for publication of this case report and accompanying images. A copy of the written consent is available for review by the Editor-in-Chief of this journal.
